# Transcriptome and Metabolome Analyses Reveal a Complex Stigma Microenvironment for Pollen Tube Growth in Tobacco

**DOI:** 10.3390/ijms252212255

**Published:** 2024-11-14

**Authors:** Hanxian Xiong, Junjie Wang, Xiaodi Gao, Guoqing Dong, Wanyong Zeng, Wei Wang, Meng-Xiang Sun

**Affiliations:** 1 School of Life Science and Technology, Wuhan Polytechnic University, Wuhan 430023, China; xionghx@whpu.edu.cn (H.X.); wjjwhpu@163.com (J.W.); gaoxiaodi5311@163.com (X.G.); donggq@whpu.edu.cn (G.D.); vyzeng@163.com (W.Z.); 2 State Key Laboratory of Hybrid Rice, College of Life Sciences, Wuhan University, Wuhan 430072, China; mxsun@whu.edu.cn

**Keywords:** microenvironment, pollen tube growth, stigma, metabolome, transcriptome, cis-zeatin riboside, tobacco

## Abstract

In flowering plants, the success of fertilization depends on the rapid polar extension of a pollen tube, which delivers sperm cells to the female gametophyte for fertilization. Numerous studies have shown that the microenvironment in planta is more conducive to the growth and development of pollen tubes than that in vitro. However, how stigma factors coordinate to regulate pollen tube growth is still poorly understood. Here, we demonstrate that in tobacco, mature stigma extract, but not immature stigma extract, facilitates pollen tube growth. Comparative transcriptomic and qRT-PCR analyses showed that the differentially expressed genes during stigma maturation were mainly enriched in the metabolism pathway. Through metabolome analyses, about 500 metabolites were identified to be differently accumulated; the significantly increased metabolites in the mature stigmas mainly belonged to alkaloids, flavonoids, and terpenoids, while the downregulated differential metabolites were related to lipids, amino acids, and their derivatives. Among the different kinds of plant hormones, the cis-form contents of zeatin were significantly increased, and more importantly, cis-zeatin riboside promoted pollen tube growth in vitro. Thus, our results reveal an overall landscape of gene expression and a detailed nutritional microenvironment established for pollen tube growth during the process of stigma maturation, which provides valuable clues for optimizing in vitro pollen growth and investigating the pollen–stigma interaction.

## 1. Introduction

During the sexual reproduction of angiosperms, after a pollen grain lands on the top of the stigma, it forms a tubular protrusion that navigates through the female tissues of the pistil, enters the ovules, and delivers its sperm cell cargo to induce fertilization [1,2]. Therefore, pollen tube growth is an indispensable event in the double fertilization of flowering plants. Besides their fundamental role in fertilization, pollen tubes also represent outstanding systems for studying the dynamics and spatiotemporal control of polarized cell growth owing to their unique characteristics, which include fast tip growth, easy culture in vitro, and a haploid genome [3].

The pollen tube is a highly specialized polar structure, with a proximal streaming region and a distal region of highly vacuolated cytoplasm, which are separated by callose plugs. Thus, the cytoplasm remains in the front portion of the pollen tube regardless of the pollen tube’s growth. The streaming region is subdivided into the shank region, which contains the two sperm cells and the vegetative nucleus; the sub-apical organelle-rich region, which contains the endoplasmic reticulum, Golgi apparatus, and mitochondria; and the tip region, with an inverted cone shape, characterized by the presence of an abundant number of transport vesicles [4,5]. The tip growth of a pollen tube requires active exo/endocytosis processes, cell wall deposition and recycling, and a highly dynamic cytoskeleton, responsible for the polar organization of the pollen tube cytoplasm and targeted exocytosis [4,6,7,8,9,10,11,12]. At the molecular level, pollen tube tip growth depends on an ROP GTPase-dependent signaling network, ROS, cellular pH, ions, etc. [4,13,14,15,16].

Although pollen grains can germinate and form pollen tubes in vitro, numerous studies have shown that the in planta pollen germination rates, pollen tube growth rates, and final tube lengths are far greater than those observed in vitro, pointing to the fact that the pistil can create a more favorable microenvironment for pollen tube germination and growth [17,18,19,20,21,22]. Thus, besides providing essential and indispensable elements, the pistil may also produce stimulants to promote pollen germination and pollen tube growth. Until now, the pistil factors known to affect pollen tube growth are proteins, plant hormones, and small organic and inorganic molecules, e.g., transmitting-tissue-specific glycoproteins [23], γ-aminobutyric acid [24,25], D-serine [26], long-chain base phosphates [27], azadecalins [28], brassinosteroids [29], STIL (Style Interactor for LePRKs) [30], hydrogen peroxide [31,32], chemocyanin, and kaempferol [33,34]. However, there is no clear picture of which elements are involved in the construction of the pistil microenvironment and how these pistil factors regulate pollen tube growth [35,36,37,38,39,40,41,42]. There is no doubt that identification and analysis of the microenvironment of a pollen tube’s growth in vivo would provide its overall landscape, thus facilitating molecular–genetic dissections of the pathways through which pistil components positively contribute to pollen tube growth. Here, we show that in tobacco, mature stigma extract stimulated pollen tube growth. Transcriptomic and metabolome analyses revealed a huge difference in the metabolites between immature and mature stigmas. The metabolites significantly upregulated in mature stigmas are mainly alkaloids, flavonoids, and terpenoids. Moreover, a sharp increase in the content of cis-zeatin riboside was observed in the mature stigmas, and in vitro pollen germination assays showed that cis-zeatin riboside contributed to pollen tube growth. Thus, our results may help to establish a foundation for further research investigating the regulatory networks of the interaction between the pollen and stigma during the pollen tube journey in a pistil.

## 2. Results

### 2.1. Mature Stigma Extract Stimulates Tobacco Pollen Tube Growth

**Figure 1:**
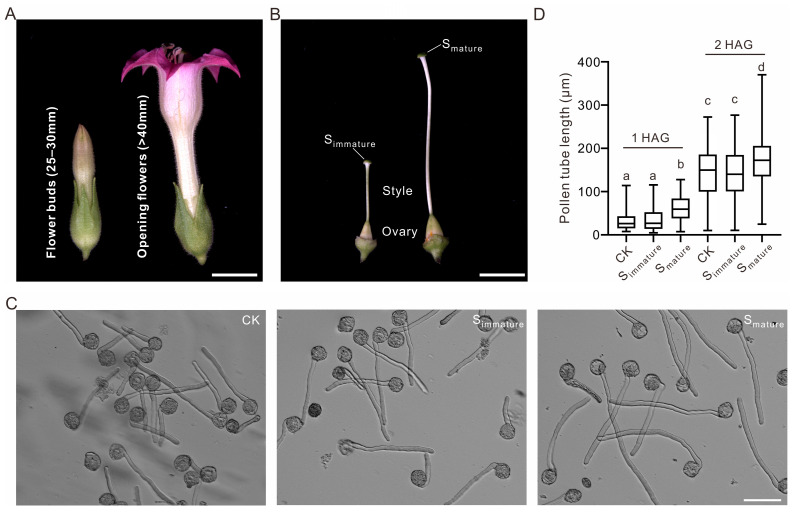
Mature stigma extract promotes pollen tube growth in tobacco. (**A**) Representative images of tobacco flower buds at 25–30 mm and at the anther dehiscence stage. (**B**) Representative images of pistils from tobacco flower buds (25–30 mm) and opening flowers. S_immature_, stigma at flower buds (25–30 mm); S_mature_, stigma at opening flowers. Bars = 10 mm. (**C**) Images of pollen germinated with S_immature_ or S_mature_ extracts at 2 h after germination (HAG). Without stigma extracts as the control (CK). Bar = 100 μm. (**D**) Statistical data of wild-type pollen tube length at 1 or 2 HAG, with and without stigma extracts. The data for pollen tube length are presented in the box-and-whisker plots. The centerline in the plot represents the 50th percentile. The bottom and top of each box indicate the 25th and 75th percentiles, respectively, and the whiskers represent the minimum and maximum values. For 1 HAG, the number of pollen tubes in CK, S_immature_, and S_mature_ are 245, 255, and 254, respectively. For 2 HAG, the number of pollen tubes in CK, S_immature_, and S_mature_ are 252, 231, and 236, respectively. For statistical comparisons of the multiple groups in (**D**), different letters denote that these groups differ significantly (*p* < 0.0001) in one-way ANOVA and Tukey’s range test.

To examine whether the stigma—the first female tissue that pollen makes contact with—can stimulate pollen tube growth, we chose tobacco stigmas as our experimental material because of their relatively large size, which is convenient for sampling and subsequent omics analysis. Immature stigmas (henceforth referred to as S_immature_) correspond to flower buds at 25–30 mm, and mature stigmas (henceforth referred to as S_mature_) correspond to the flower opening stage (>40 mm) (Figure 1A,B). At 1 and 2 h after germination, the pollen with an addition of S_mature_ extract germinated to a significantly longer tube length than that without, indicating that the mature stigma extract promoted pollen tube growth (Figure 1C,D). Additionally, there was no stimulation of pollen tube growth when using S_immature_, indicating that the germination stimulant(s) was either abundant or specifically present in the mature stigmas (Figure 1C,D). Therefore, in tobacco, the microenvironment in S_mature_ is more beneficial to pollen tube growth than that in S_immature_.

### 2.2. Transcriptome Changes Between Immature and Mature Stigmas

**Figure 2:**
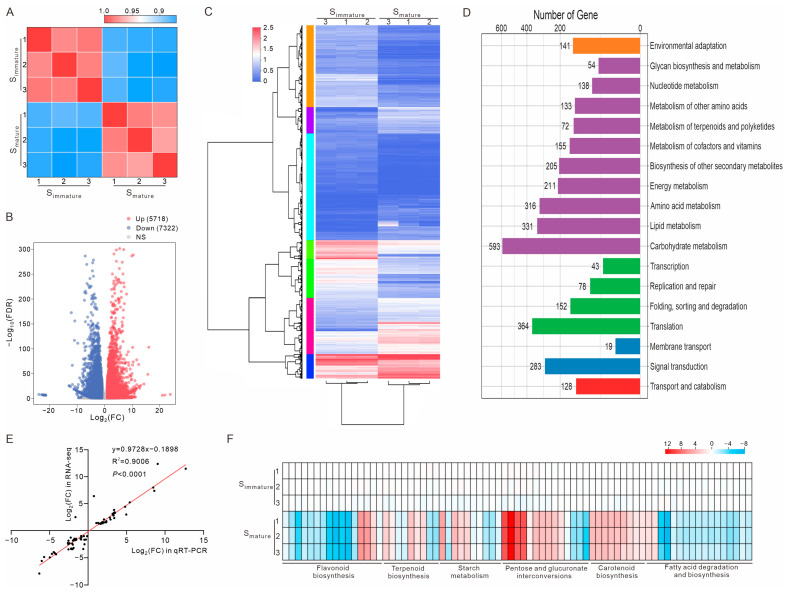
Comparative transcriptomic analysis of immature and mature stigmas. (**A**) Heat map showing Pearson’s correlation across RNA-seq datasets. Three biological replicates for each sample. (**B**) Volcano plot depicts differentially expressed genes between S_immature_ and S_mature_. Red dots indicate upregulated genes, while blue dots indicate downregulated genes. FC, fold change; FDR, false discovery rate; NS, not significant. (**C**) Hierarchical clustering of differentially expressed genes (DEGs). DEGs between S_immature_ and S_mature_ could be categorized into seven distinct clusters. (**D**) KEGG pathway analysis of DEGs. The number of enriched genes is shown above the column. (**E**) High correlation between quantitative real-time PCR (qRT-PCR) and RNA sequencing results (75 genes). (**F**) Verification of DEGs in flavonoid biosynthesis, terpenoid biosynthesis, starch and sucrose metabolism, pentose and glucuronate interconversions, carotenoid biosynthesis, and fatty acid degradation and biosynthesis pathways using qRT-PCR. The expression level of each gene was estimated using log_2_ (FC).

To understand how gene expression impacts the microenvironment of pollen tube growth in stigmas, RNA sequencing of S_immature_ and S_mature_ was performed. Summaries of the sequencing samples and results are shown in the Supplementary Data S1. After removing low-quality reads, approximately 22 million clean reads were obtained for each biological replicate. Overall, the transcripts from the same cell type but across different biological replicates exhibited a high correlation (r > 0.96) (Figure 2A). A total of 13,040 (5718 upregulated and 7322 downregulated) differentially expressed genes (DEGs) were identified between S_immature_ and S_mature_ (Figure 2B and Supplementary Data S2 and S3). A hierarchical clustering analysis of the DEGs is shown with a heat map; the DEGs were clustered into seven groups based on their abundance changes (Figure 2C and Figure S1). These results show that the expression patterns of the DEGs were significantly different between S_immature_ and S_mature_. Compared to S_immature_, the expression levels of approximately 15% of the genome genes were changed in S_mature_, indicative of huge physiological and biochemical changes during stigma maturation. Next, these DEGs were subjected to a KEGG pathway analysis, and the results show that the pathways related to transport and catabolism, transcription and translation, and signal transduction were enriched (Figure 2D and Figures S2 and S3). It is noteworthy that most DEGs were enriched in metabolic pathways, such as carbohydrates, energy, lipids, amino acids, terpenoids and polyketides, cofactors and vitamins, flavonoids, and phenylpropanoids, indicative of their potential roles as members of the microenvironment to promote pollen tube growth.

To validate the accuracy of the RNA sequencing data, 75 DEGs related to the metabolism pathway were selected for quantitative real-time PCR (qRT-PCR) analysis. The fold changes of 74 DEGs obtained from the qRT-PCR analysis were consistent with those obtained from the transcriptome analysis, and a significant positive correlation between the RNA-seq and qRT-PCR results was observed (Figure 2E,F). These results verify the accuracy of the RNA sequencing data and indicate that the expression levels of genes involved in the metabolic pathway, such as flavonoid biosynthesis, terpenoid biosynthesis, starch and sucrose metabolism, pentose and glucuronate interconversions, carotenoid biosynthesis, and fatty acid degradation and biosynthesis, were truly changed.

### 2.3. Metabolite Profiling Between Immature and Mature Stigmas

**Figure 3:**
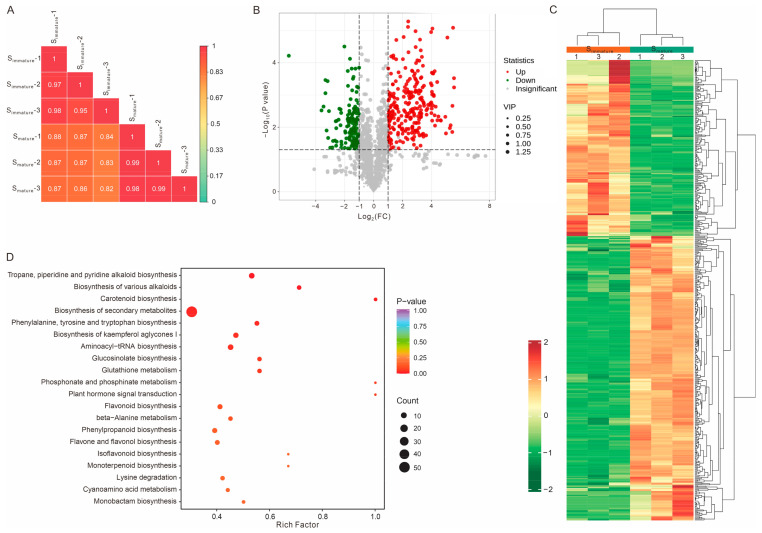
Determination of significantly changed metabolites between immature and mature stigmas. (**A**) Pearson’s correlation of all six stigma samples. (**B**) A volcano map of differentially regulated metabolites between S_immature_ and S_mature_. Red and green dots represent significantly up- or downregulated metabolites, respectively. The dot size indicates the VIP (variable importance in projection) values. FC, fold change. (**C**,**D**) Heat map clustering and KEGG enrichment of differentially regulated metabolites between S_immature_ and S_mature_.

Metabolomics analysis can enrich and further verify the accuracy of transcriptomic sequencing results; therefore, a comprehensive metabolic profiling of S_immature_ and S_mature_ was performed. A total of 1635 metabolites were identified from the stigma tissue, which could be further classified into thirteen categories (Figure S4 and Supplementary Data S4) as follows: alkaloids (14.01%), amino acids and derivatives (13.76%), lipids (12.78%), flavonoids (12.35%), terpenoids (9.66%), phenolic acids (7.95%), organic acids (5.5%), nucleotides and derivatives (4.89%), lignans and coumarins (3.18%), quinones (0.98%), tannins (0.18%), steroids (0.12%), and others (14.62%). Next, we focused on the differentially regulated metabolites between the immature and mature stigmas. Compared to S_immature_, there were 298 differentially upregulated and 185 differentially downregulated metabolites in S_mature_ (Figure 3A–C and Supplementary Data S5). The significantly upregulated metabolites mainly belonged to alkaloids, flavonoids, and terpenoids, whereas the downregulated differential metabolites were mainly lipids, amino acids, and their derivatives (Figure S5 and Supplementary Data S5). Moreover, the top ten upregulated metabolites comprised eight alkaloids and two flavones (fold change > 50) (Figure S6). Conjoint transcriptome and metabolome analysis revealed that the DEGs and different metabolites could be enriched in flavonoid, flavone, and flavonol biosynthesis pathways, indicating that flavonoids might have an important role in promoting pollen tube growth (Figures S7 and S8). Previous studies have shown that flavonoids are required for pollen vitality, germination, and pollen tube development [33,43,44,45,46,47,48,49,50,51], and several flavonols (quercetin, kaempferol, and myricetin) have strong stimulatory effects on tobacco pollen germination frequency and pollen tube length in vitro [33,34]. Thus, these upregulated flavonols, other upregulated compounds (alkaloids and terpenoids), or a combination may be required for the stigma microenvironment to support rapid pollen tube growth.

### 2.4. Cis-Zeatin Riboside Facilitates Tobacco Pollen Tube Growth In Vitro

**Figure 4:**
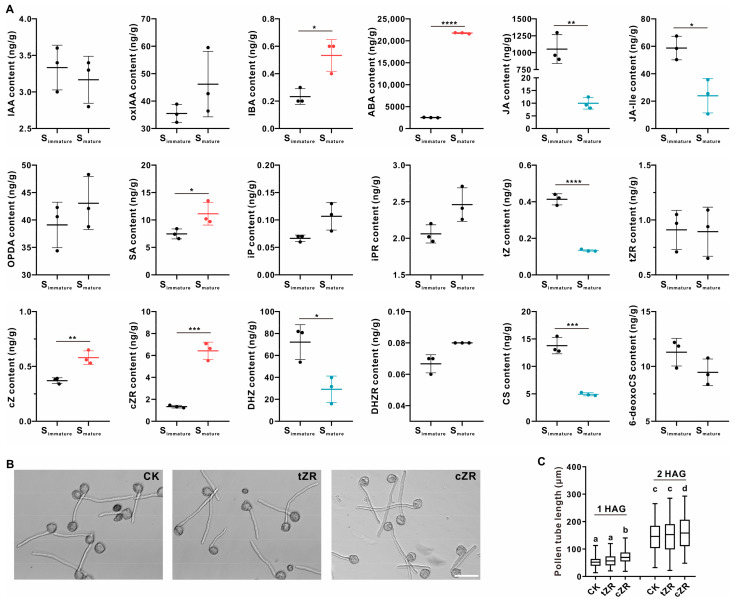
cZR promotes tobacco pollen tube growth in vitro. (**A**) The quantification of different kinds of plant hormones in S_immature_ and S_mature_. The data are presented as the mean ± SD from three independent replicates. The up-regulated hormones were indicated in red and the down-regulated shown in blue. Two-tailed Student’s *t*-test was used for statistical analysis (* *p* < 0.05; ** *p* < 0.01; *** *p* < 0.001; **** *p* < 0.0001). IAA, 3-indolylacetic acid; oxIAA, 2-oxo-3-indolineacetic acid; IBA, 4-(3-indolyl)butyric acid; ABA, abscisic acid; JA, jasmonic acid; JA-Ile, jasmonoyl-L-isoleucine; OPDA, 12-oxo-phytodienoic acid; SA, salicylic acid; iP, N6-isopentenyladenine; iPR, N6-isopentenyladenosine; tZ, trans-zeatin; tZR, trans-zeatin riboside; cZ, cis-zeatin; cZR, cis-zeatin riboside; DHZ, dihydrozeatin; DHZR, dihydrozeatin riboside; CS, castasterone; 6-deoxoCS, 6-deoxocastasterone. (**B**) Images of pollen germinated with supplement of tZR or cZR at 2 h after germination (HAG). Without the addition of tZR or cZR as a control (CK). Bar = 100 μm. (**C**) Statistical data of wild-type pollen tube length at 1 or 2 HAG. The data for the pollen tube length are presented in the box-and-whisker plots. The centerline in the plot represents the 50th percentile. The bottom and top of each box indicate the 25th and 75th percentiles, respectively, and the whiskers represent the minimum and maximum values. For 1 HAG, the number of pollen tubes in CK, tZR, and cZR is 173, 191, and 197, respectively. For 2 HAG, the number of pollen tubes in CK, tZR, and cZR is 194, 216, and 173, respectively. Ordinary one-way ANOVA with Tukey’s multiple comparisons test was used for statistical difference analysis among different groups. Different letters above the bars indicate significant differences (*p* < 0.05) among the groups.

Plant hormones are also factors known to affect pollen tube growth and could be part of the microenvironment beneficial to pollen tube growth. Previous studies have shown that auxin, gibberellins, jasmonic acids (JAs), and brassinosteroids promote in vitro pollen germination and growth, whereas abscisic acid shows an inhibiting effect [52,53,54,55,56,57,58]. We analyzed different kinds of endogenous plant hormones, including their derivatives or precursors in tobacco stigmas. Although indole-3-butyric acid (IBA), an auxin precursor, was upregulated in mature stigmas, no significant changes in the IAA levels nor oxIAA (oxidation of IAA) levels were observed (Figure 4A). Compared to S_immature_, abscisic acid and salicylic acid were significantly upregulated in S_mature_, while the JA and JA-Ile contents showed the opposite trend (Figure 4A and Figure S9). These results indicate that at least in tobacco stigmas, auxin and jasmonic acids are not the active compounds that promote pollen tube growth. Gibberellins and brassinosteroids could not be ruled out, as their concentrations in the stigmas were below the limit of detection.

Typical representatives of isoprenoid cytokinins are isopentenyl adenine (iP) and its hydroxylated forms zeatin (Z) and dihydrozeatin (DHZ) [59]. Our results reveal that the content of DHZ decreased in the mature stigmas, but the iP level showed no change. There are different zeatin isomers—cis-zeatin (cZ) and trans-zeatin (tZ), and their ribosides. The concentrations of tZ and cZ show the opposite trend: the tZ content decreased during the two stages of the investigation, whereas the contents of cZ and cis-zeatin riboside (cZR) increased, especially the latter, by up to three times (Figure 4A). Therefore, we investigated whether cZR was involved in promoting pollen tube growth. In vitro pollen gemination assays proved that the addition of cZR enhanced the pollen tube’s growth, while a supplement of tZR did not (Figure 4B,C). Therefore, cZR is a candidate stimulant for pollen tube growth in tobacco stigmas.

## 3. Discussion

In flowering plants, pollen tubes often elongate by hundreds to thousands of times the length of the pollen grains to reach the ovary. In tobacco, this distance is usually more than 30 mm [54]. To guarantee successful fertilization, enough pollen tubes need to arrive at the ovary to fertilize most of the ovules before stylar abscission (around 72 h after pollination). Therefore, in vivo pistils have evolved a microenvironment to accelerate pollen tube growth to ensure rapid fertilization within the narrow developmental window during which ovules are receptive. Using an in vitro pollen germination bioassay, we defined a role for tobacco stigmas in promoting pollen tube growth, finding that the microenvironment in mature stigmas was more favorable to pollen tube elongation.

Angiosperm stigmas can be classified into two broad categories—wet and dry. *Nicotiana* belongs to the latter and possesses a surface secretion in its stigmas [60]. It was found that the components of the stigma secretion of *Nicotiana*, rather than the stigma cells themselves, were necessary and sufficient for compatible pollen germination and pollen tube growth [61,62]. Besides serving as a storage site for pollen hydration and pollen tube growth [60,63,64], tobacco stigmas also contain significant amounts of hydrogen peroxide, influencing pollen tube membrane hyperpolarization and changing the pollen proteome [31], which was later proven to stimulate pollen germination and ensure successful fertilization in vivo [32]. However, until now, few detailed studies of the pollen microenvironment in stigmas have been conducted [35,36,37,38,39,40,41,42]. Here, both transcriptome and metabolome analyses at two stages of stigma development were carried out, pointing to huge differences in the accumulation of metabolites between S_immature_ and S_mature_. The fact that 15% of the genome genes were changed and that about 300 metabolites, including several plant hormones, were upregulated in mature stigmas, indicates a complex stigma microenvironment for pollen tube growth in tobacco. In mature stigmas, there are three main categories of upregulated metabolites, including alkaloids, flavonoids, and terpenoids. Although flavonoids and terpenoids are derived from different metabolic pathways, they are metabolically linked [65,66,67,68], and both are closely related to pollen–stigma interactions [33,43,49,69]. A very challenging but important future task will be to determine their biological function in plant fertilization, perhaps not limited to promoting pollen tube growth but also including pollen tube penetration or guidance.

So far, chemical substances that have been identified to be produced by pistil tissues and to stimulate in vitro pollen germination or pollen tube growth are proteins and small organic or inorganic molecules [23,24,25,26,27,28,29,30,31,32,33]. In addition to these, plant hormones (auxin, gibberellic acid, brassinosteroids, and jasmonic acid) have also been reported to promote pollen germination and growth in *Prunus avium* [52], *Torenia fournieri* [55,56], tobacco [54], and *Arabidopsis* [57,58]. Here, we found that cis-zeatin riboside is a new stimulator of pollen tube growth from the stigma. Zeatin was first discovered in maize (*Zea mays*) [70] and belongs to an important class of plant hormones called cytokinins, which regulate many physiological processes, including cell proliferation and differentiation, various aspects of shoot and root growth and development, and stress resilience [59,71,72,73,74]. Zeatin is widespread in nature [75] and can be distinguished as two geometrical isomers denoted as cis- and trans-zeatin. While cis-forms have long been considered less important than trans-forms due to low attributed biological activity, several studies have suggested that cis-forms play different roles from the trans-forms in plant development and adaptation to stress [76,77,78,79,80,81]. In this study, we found that cis-forms were upregulated in the mature stigmas and able to stimulate tobacco pollen tube growth, whereas the trans-form were not, indicating that cis-forms have unique physiological functions in regulating pollen tube growth. In plants, both pollen tubes and root hairs serve as excellent model systems for rapid polar tip growth. In the Pi starvation response (PSR), tZ preferentially represses cell cycle and PSR genes, whereas cZ induces genes involved in cell and root hair elongation and differentiation [82]. More importantly, cZ acts as a PSR hormone that stimulates root hair elongation. Together with our results, these findings support the role of cis-forms in regulating polarity growth [82]. Cytokinins are mobile signals, and we speculated that they can be translocated from stigmas to pollen to promote pollen tube growth. In *Arabidopsis*, two isopentenyl transferase enzymes (IPT2 and IPT9) are responsible for cZ-type production following tRNA degradation [83]. It is worth knocking out the homologous genes in the tobacco and investigating whether the wild-type pollen tube growth rate is reduced or impaired in the mutant stigma.

It has been shown that the substances that the pistil tissues release to accelerate pollen tube growth are diversified in plants [23,24,25,26,27,28,29,30,31,32,33,34]. Meanwhile, the activity of pollen stimulants seems to be species-specific. Flavonols stimulate pollen germination and growth in petunia, tobacco, and tomato but are not required for *Arabidopsis* pollen germination [33,34,84,85]. Sulfinylated azadecalins could stimulate pollen germination in three species of Lineage I of Brassicaceae but do not induce a germination response in *Sisymbrium irio* (Lineage II of Brassicaceae) and tobacco [28]. And, given the variety of stigma structures and microecology in flowering plants, identification of the role of the stigma in pollen tube growth and analyses of the stigma microenvironment in more plants is very necessary and will be no doubt update our current understanding of the molecular identity of exchange signals between pollen and stigmas, and the cellular interactions that they regulate.

## 4. Materials and Methods

### 4.1. Plant Materials

Tobacco (*Nicotiana tabacum* L. cv. Petite Havana SR1) plants were grown under 12 h of daylight at 25 °C in a glasshouse. Tobacco pollen was germinated in a pollen liquid germination medium (PGM) (5 µM CaCl_2_, 5 µM Ca(NO3)_2_, 1 mM MgSO4, 0.01% H_3_BO_3_, and 18% sucrose) under 28 °C in the dark for 1 h and 2 h. For the treatment of the immature stigmas, the pistils were dissected from the flower buds at 25–30 mm; for the mature stigmas, the flowers (>35 mm but unflowering) were emasculated 24 h before dissection. Then, the pistils were cut at the junction between the stigma and the style. Four stigmas (for each experiment) were ground in liquid nitrogen and dissolved in 1 mL PGM, which was then filtered to obtain a PGM containing stigma extract. Here, PGM without stigma extract was used as the control. For the treatment of zeatin, final concentrations of PGM containing 2.5 µM trans-zeatin riboside and cis-zeatin riboside (Sangon Biotech, Shanghai, China) were prepared by adding 1 µL stock solution (2.5 mM) dissolved in DMSO to 1.0 mL PGM. Here, PGM with 0.1% DMSO was used as the control.

### 4.2. RNA Extraction, Transcriptome Sequencing, and Quantitative Real-Time PCR (qRT-PCR)

The total RNA was extracted using the TaKaRa MiniBEST Plant RNA Extraction Kit (Takara, Beijing, China) following the manufacturer’s protocol. For S_immature_ and S_mature_, 10 stigmas were used. After the RNA extraction, the high-quality total RNA was sent to Frasergen (Wuhan, China) for cDNA library construction. Then, the libraries were sequenced on an MGI-SEQ 2000 platform by Frasergen Bioinformatics Co., Ltd., Wuhan, China. After data filtering, the clean reads were mapped to the tobacco reference genome using HISAT2 2.2.1 software with the default parameters [86,87,88]. For each transcript, FPKM (fragments per kilobase of transcript per million mapped reads) were calculated using RSEM 1.3.3 [89]. The differentially expressed genes (DEGs) were identified using DESeq2 1.22.2 software [90] with a |log_2_ (fold change)| ≥ 1 and false discovery rate (FDR) < 0.05. Next, the Kyoto Encyclopedia of Genes and Genomes (KEGG) tools were used to annotate the potential metabolic pathways of the DEGs [91,92,93].

The cDNA was synthesized using a HiScript II 1st Strand cDNA Synthesis Kit (Vazyme, Nanjing, China). qRT-PCR was performed according to a previously reported method [94]; the primers used are listed in the Supplementary Data S6.

### 4.3. Metabolomics Analysis

Tobacco stigmas (500 mg) were collected, and sample extraction and metabolomics analysis were subsequently performed at Wuhan MetWare Biotechnology Co., Ltd., Wuhan, China (www.metware.cn) according to their standard procedures [95,96]. The sample extracts were analyzed using a UPLC-ESI-MS/MS system (UPLC, Chicago, IL, USA, ExionLC™ AD https://sciex.com.cn/) and tandem mass spectrometry system (https://sciex.com.cn/). Metabolite quantification was performed using the multiple reaction monitoring (MRM) mode in a triple/quadrupole mass spectrometer [96]. The variable importance in projection (VIP) score obtained from the OPLS-DA (version 1.0.1) results and metabolites with both VIP > 1 and |log_2_ (fold change)| ≥ 1 were determined as significantly changed metabolites [97,98].

### 4.4. Hormones Measurements

Tobacco stigmas (300 mg) were collected and sent to the Wuhan Greensword Creation Technology Co. Ltd., (Wuhan, China) (http://www.greenswordcreation.com). Analysis of the phytohormones was performed on a UHPLC-MS/MS system (Thermo Scientific Ultimate 3000 UHPLC coupled with TSQ Quantiva, Waltham, MA, USA) according to a previous protocol [99].

### 4.5. Statistical Analysis

Statistical analyses were performed using GraphPad Prism software (version 8.0). Statistical significance was determined using two-sided Student’s *t*-test or the Tukey–Kramer multiple comparison test.

## 5. Conclusions

In summary, our study determined the pollen tube growth-promoting properties of tobacco stigmas, which depend, at least partly, on cis-zeatin compounds. The diversity of the endogenous factors that accelerate pollen tube growth and the findings that up to 300 metabolites were upregulated in the mature stigmas support the notion that multiple factors work together to boost pollen tube growth for rapid double fertilization. This issue needs to be explored in the future; perhaps we can create an active microenvironment in vitro similar to that in planta using a combination of different stigma factors after sufficient growth-promoting substances have been identified in future studies.

## Data Availability

The data are contained within the article.

## Supplementary Materials

The following supporting information can be downloaded at https://www.mdpi.com/article/10.3390/ijms252212255/s1.

## References

[1] Dresselhaus T., Franklin-Tong N. (2013). Male-female crosstalk during pollen germination, tube growth and guidance, and double fertilization. Mol. Plant.

[2] Sprunck S. (2020). Twice the fun, double the trouble: Gamete interactions in flowering plants. Curr. Opin. Plant Biol..

[3] Qin Y., Yang Z.B. (2011). Rapid tip growth: Insights from pollen tubes. Semin. Cell Dev. Biol..

[4] Guan Y.F., Guo J.Z., Li H., Yang Z.B. (2013). Signaling in pollen tube growth: Crosstalk, feedback, and missing links. Mol. Plant.

[5] Franklin-Tong V.E. (1999). Signaling and the modulation of pollen tube growth. Plant Cell.

[6] Yang Z.B. (2008). Cell polarity signaling in *Arabidopsis*. Annu. Rev. Cell Dev. Biol..

[7] Cheung A.Y., Wu H.M. (2008). Structural and signaling networks for the polar cell growth machinery in pollen tubes. Ann. Rev. Plant Biol..

[8] Qu X.L., Jiang Y.X., Chang M., Liu X.N., Zhang R.H., Huang S.J. (2015). Organization and regulation of the actin cytoskeleton in the pollen tube. Front. Plant Sci..

[9] Zhang Y., McCormick S. (2010). The regulation of vesicle trafficking by small GTPases and phospholipids during pollen tube growth. Sex Plant Reprod..

[10] Hafidh S., Honys D. (2021). Reproduction multitasking: The male gametophyte. Annu. Rev. Plant Biol..

[11] Ruan H.Q., Li J., Wang T., Ren H.Y. (2021). Secretory vesicles targeted to plasma membrane during pollen germination and tube growth. Front Cell Dev. Biol..

[12] Hao G.J., Zhao X.Y., Zhang M.M., Ying J., Yu F., Li S., Zhang Y. (2022). Vesicle trafficking in *Arabidopsis* pollen tubes. FEBS Lett..

[13] Zhang M.J., Zhang X.S., Gao X.Q. (2020). ROS in the male-female interactions during pollination: Function and regulation. Front. Plant Sci..

[14] Diao M., Qu X.L., Huang S.J. (2018). Calcium imaging in pollen cells using G-CaMP5. J. Integr Plant Biol..

[15] Zhou Z.G., Zheng S., Ul Haq S.I., Zheng D.F., Qiu Q.S. (2022). Regulation of pollen tube growth by cellular pH and ions. J. Plant Physiol..

[16] Li E., Zhang Y.L., Qin Z., Xu M., Qiao Q., Li S., Li S.W., Zhang Y. (2023). Signaling network controlling ROP-mediated tip growth in *Arabidopsis* and beyond. Plant Comm..

[17] Rodriguez-Enriquez M.J., Mehdi S., Dickinson H.G., Grant-Downton R.T. (2013). A novel method for efficient germination and tube growth of *Arabidopsis thaliana* pollen. New Phytol..

[18] Mouline K., Véry A.A., Gaymard F., Boucherez J., Pilot G., Devic M., Bouchez D., Thibaud J.B., Sentenac H. (2002). Pollen tube development and competitive ability are impaired by disruption of a Shaker K(+) channel in *Arabidopsis*. Gene Dev..

[19] Li H., Lin Y.K., Heath R.M., Zhu M.X., Yang Z.B. (1999). Control of pollen tube tip growth by a Rop GTPase-dependent pathway that leads to tip-localized calcium influx. Plant Cell.

[20] Fan L.M., Wang Y.F., Wang H., Wu W.H. (2001). In vitro *Arabidopsis* pollen germination and characterization of the inward potassium currents in pollen grain protoplasts. J. Exp. Bot..

[21] Mayfield J.A., Preuss D. (2000). Rapid initiation of *Arabidopsis* pollination requires the oleosin-domain protein GRP17. Nat. Cell Biol..

[22] Johnson M.A., Preuss D. (2002). Plotting a course: Multiple signals guide pollen tubes to their targets. Dev. Cell.

[23] Cheung A.Y., Wang H., Wu H.M. (1995). A floral transmitting tissue-specific glycoprotein attracts pollen tubes and stimulates their growth. Cell.

[24] Palanivelu R., Brass L., Edlund A.F., Preuss D. (2003). Pollen tube growth and guidance is regulated by POP2, an gene that controls GABA levels. Cell.

[25] Yu G.H., Zou J., Feng J., Peng X.B., Wu J.Y., Wu Y.L., Palanivelu R., Sun M.X. (2014). Exogenous γ-aminobutyric acid (GABA) affects pollen tube growth via modulating putative Ca^2+^-permeable membrane channels and is coupled to negative regulation on glutamate decarboxylase. J. Exp. Bot..

[26] Michard E., Lima P.T., Borges F., Silva A.C., Portes M.T., Carvalho J.E., Gilliham M., Liu L.H., Obermeyer G., Feijó J.A. (2011). Glutamate receptor-like genes form Ca^2+^ channels in pollen tubes and are regulated by pistil D-serine. Science.

[27] Wu J.Y., Qin X.Y., Tao S.T., Jiang X.T., Liang Y.K., Zhang S.L. (2014). Long-chain base phosphates modulate pollen tube growth via channel-mediated influx of calcium. Plant J..

[28] Qin Y., Wysocki R.J., Somogyi A., Feinstein Y., Franco J.Y., Tsukamoto T., Dunatunga D., Levy C., Smith S., Simpson R. (2011). Sulfinylated azadecalins act as functional mimics of a pollen germination stimulant in *Arabidopsis* pistils. Plant J..

[29] Vogler F., Schmalzl C., Englhart M., Bircheneder M., Sprunck S. (2014). Brassinosteroids promote *Arabidopsis* pollen germination and growth. Plant Reprod..

[30] Wengier D.L., Mazzella M.A., Salem T.M., McCormick S., Muschietti J.P. (2010). STIL, a peculiar molecule from styles, specifically dephosphorylates the pollen receptor kinase LePRK2 and stimulates pollen tube growth in vitro. BMC Plant Biol..

[31] Breygina M., Klimenko E., Shilov E., Podolyan A., Mamaeva A., Zgoda V., Fesenko I. (2021). Hydrogen peroxide in tobacco stigma exudate affects pollen proteome and membrane potential in pollen tubes. Plant Biol..

[32] Breygina M., Schekaleva O., Klimenko E., Luneva O. (2022). The balance between different ROS on tobacco stigma during flowering and its role in pollen germination. Plants.

[33] Mo Y.Y., Nagel C., Taylor L.P. (1992). Biochemical complementation of chalcone synthase mutants defines a role for flavonols in functional pollen. Proc. Natl. Acad. Sci. USA.

[34] Ylstra B., Touraev A., Moreno R.M.B., Stoger E., Vantunen A.J., Vicente O., Mol J.N.M., Heberlebors E. (1992). Flavonols stimulate development, germination, and tube growth of tobacco pollen. Plant Physiol..

[35] Sang Y.L., Xu M., Ma F.F., Chen H., Xu X.H., Gao X.Q., Zhang X.S. (2012). Comparative proteomic analysis reveals similar and distinct features of proteins in dry and wet stigmas. Proteomics.

[36] Quiapim A.C., Brito M.S., Bernardes L.A.S., daSilva I., Malavazi I., DePaoli H.C., Molfetta-Machado J.B., Giuliatti S., Goldman G.H., Goldman M.H.S. (2009). Analysis of the *Nicotiana tabacum* stigma/style transcriptome reveals gene expression differences between wet and dry stigma species. Plant Physiol..

[37] Swanson R., Clark T., Preuss D. (2005). Expression profiling of *Arabidopsis* stigma tissue identifies stigma-specific genes. Sex. Plant Reprod..

[38] Tung C.W., Dwyer K.G., Nasrallah M.E., Nasrallah J.B. (2005). Genome-wide identification of genes expressed in *Arabidopsis* pistils specifically along the path of pollen tube growth. Plant Physiol..

[39] Yue X., Gao X.Q., Wang F., Dong Y.X., Li X.G., Zhang X.S. (2014). Transcriptional evidence for inferred pattern of pollen tube-stigma metabolic coupling during pollination. PLoS ONE.

[40] Matsuda T., Matsushima M., Nabemoto M., Osaka M., Sakazono S., Masuko-Suzuki H., Takahashi H., Nakazono M., Iwano M., Takayama S. (2015). Transcriptional characteristics and differences in *Arabidopsis* stigmatic papilla cells pre- and post-pollination. Plant Cell Physiol..

[41] Iwano M., Igarashi M., Tarutani Y., Kaothien-Nakayama P., Nakayama H., Moriyama H., Yakabe R., Entani T., Shimosato-Asano H., Ueki M. (2014). A pollen coat-inducible autoinhibited Ca^2+^-ATPase expressed in stigmatic papilla cells is required for compatible pollination in the Brassicaceae. Plant Cell.

[42] Gao Z., Daneva A., Salanenka Y., Van Durme M., Huysmans M., Lin Z.C., De Winter F., Vanneste S., Karimi M., Van de Velde J. (2018). KIRA1 and ORESARA1 terminate flower receptivity by promoting cell death in the stigma of *Arabidopsis*. Nat. Plants.

[43] Wu H.M., Xie D.J., Jia P.F., Tang Z.S., Shi D.Q., Shui G.H., Wang G.D., Yang W.C. (2023). Homeostasis of flavonoids and triterpenoids most likely modulates starch metabolism for pollen tube penetration in rice. Plant Biotechnol. J..

[44] Wang L.X., Lam P.Y., Lui A.C.W., Zhu F.Y., Chen M.X., Liu H.J., Zhang J.H., Lo C. (2020). Flavonoids are indispensable for complete male fertility in rice. J. Exp. Bot..

[45] Coe E.H., Mccormick S.M., Modena S.A. (1981). White pollen in maize. J. Hered..

[46] Mahajan M., Ahuja P.S., Yadav S.K. (2011). Post-transcriptional silencing of flavonol synthase mRNA in tobacco leads to fruits with arrested seed set. PLoS ONE.

[47] Muhlemann J.K., Younts T.L.B., Muday G.K. (2018). Flavonols control pollen tube growth and integrity by regulating ROS homeostasis during high-temperature stress. Proc. Natl. Acad. Sci. USA.

[48] Napoli C.A., Fahy D., Wang H.Y., Taylor L.P. (1999). white anther: A petunia mutant that abolishes pollen flavonol accumulation, induces male sterility, and is complemented by a chalcone synthase transgene. Plant Physiol..

[49] Pollak P.E., Vogt T., Mo Y.Y., Taylor L.P. (1993). Chalcone synthase and flavonol accumulation in stigmas and anthers of *Petunia hybrida*. Plant Physiol..

[50] Schijlen E.G.W.M., de Vos C.H.R., Martens S., Jonker H.H., Rosin F.M., Molthoff J.W., Tikunov Y.M., Angenent G.C., van Tunen A.J., Bovy A.G. (2007). RNA interference silencing of chalcone synthase, the first step in the flavonoid biosynthesis pathway, leads to parthenocarpic tomato fruits. Plant Physiol..

[51] Taylor L.P., Jorgensen R. (1992). Conditional male-fertility in chalcone synthase-deficient petunia. J. Hered..

[52] Hewitt F.R., Hough T., Oneill P., Sasse J.M., Williams E.G., Rowan K.S. (1985). Effect of brassinolide and other growth-regulators on the germination and growth of pollen tubes of *Prunus-avium* using a multiple hanging-drop assay. Aust. J. Plant Physiol..

[53] Singh I., Shono M. (2005). Physiological and molecular effects of 24-epibrassinolide, a brassinosteroid on thermotolerance of tomato. Plant Growth Regul..

[54] Chen D., Zhao J. (2008). Free IAA in stigmas and styles during pollen germination and pollen tube growth of *Nicotiana tabacum*. Physiol. Plant.

[55] Wu J.Z., Qin Y., Zhao J. (2008). Pollen tube growth is affected by exogenous hormones and correlated with hormone changes in styles in *Torenia fournieri* L.. Plant Growth Regul..

[56] Wu J.Z., Lin Y., Zhang X.L., Pang D.W., Zhao J. (2008). IAA stimulates pollen tube growth and mediates the modification of its wall composition and structure in *Torenia fournieri*. J. Exp. Bot..

[57] Ju Y., Guo L., Cai Q., Ma F., Zhu Q.Y., Zhang Q., Sodmergen (2016). Arabidopsis JINGUBANG is a negative regulator of pollen germination that prevents pollination in moist environments. Plant Cell.

[58] Singh D.P., Jermakow A.M., Swain S.M. (2002). Gibberellins are required for seed development and pollen tube growth in *Arabidopsis*. Plant Cell.

[59] Sakakibara H. (2006). Cytokinins: Activity, biosynthesis, and translocation. Annu. Rev. Plant Biol..

[60] Hiscock S.J., Allen A.M. (2008). Diverse cell signalling pathways regulate pollen-stigma interactions: The search for consensus. New Phytol..

[61] Wolters-Arts M., Lush W.M., Mariani C. (1998). Lipids are required for directional pollen-tube growth. Nature.

[62] Wolters-Arts M., Van der Weerd L., Van Aelst A.C., Van der Weerd J., Van As H., Mariani C. (2002). Water-conducting properties of lipids during pollen hydration. Plant Cell Environ..

[63] Konar R.N., Linskens H.F. (1966). Physiology and biochemistry of the stigmatic fluid of *Petunia hybrida*. Planta.

[64] Labarca C., Loewus F. (1972). The nutritional role of pistil exudate in pollen tube wall formation in *Lilium longiflorum*: I. Utilization of injected stigmatic exudate. Plant Physiol..

[65] Ben Zvi M.M., Shklarman E., Masci T., Kalev H., Debener T., Shafir S., Ovadis M., Vainstein A. (2012). PAP1 transcription factor enhances production of phenylpropanoid and terpenoid scent compounds in rose flowers. New Phytol..

[66] Kang J.H., McRoberts J., Shi F., Moreno J.E., Jones A.D., Howe G.A. (2014). The flavonoid biosynthetic enzyme chalcone isomerase modulates terpenoid production in glandular trichomes of tomato. Plant Physiol..

[67] Sugimoto K., Zager J.J., St Aubin B., Lange B.M., Howe G.A. (2022). Flavonoid deficiency disrupts redox homeostasis and terpenoid biosynthesis in glandular trichomes of tomato. Plant Physiol..

[68] Voo S.S., Grimes H.D., Lange B.M. (2012). Assessing the biosynthetic capabilities of secretory glands in *Citrus* peel. Plant Physiol..

[69] Xue Z.Y., Xu X., Zhou Y., Wang X.N., Zhang Y.C., Liu D., Zhao B.B., Duan L.X., Qi X.Q. (2018). Deficiency of a triterpene pathway results in humidity-sensitive genic male sterility in rice. Nat. Commun..

[70] Jameson P.E. (2023). Zeatin: The 60th anniversary of its identification. Plant Physiol..

[71] Li S.M., Zheng H.X., Zhang X.S., Sui N. (2021). Cytokinins as central regulators during plant growth and stress response. Plant Cell Rep..

[72] Svolacchia N., Sabatini S. (2023). Cytokinins. Curr. Biol..

[73] Ha S., Vankova R., Yamaguchi-Shinozaki K., Shinozaki K., Tran L.S.P. (2012). Cytokinins: Metabolism and function in plant adaptation to environmental stresses. Trends Plant Sci..

[74] Pertry I., Václavíková K., Depuydt S., Galuszka P., Spíchal L., Temmerman W., Stes E., Schmülling T., Kakimoto T., Van Montagu M.C.E. (2009). Identification of *Rhodococcus fascians* cytokinins and their modus operandi to reshape the plant. Proc. Natl. Acad. Sci. USA.

[75] Gajdosová S., Spíchal L., Kamínek M., Hoyerová K., Novák O., Dobrev P.I., Galuszka P., Klíma P., Gaudinova A., Zizková E. (2011). Distribution, biological activities, metabolism, and the conceivable function of cis-zeatin-type cytokinins in plants. J. Exp. Bot..

[76] Köllmer I., Novák O., Strnad M., Schmülling T., Werner T. (2014). Overexpression of the cytosolic cytokinin oxidase/dehydrogenase (CKX7) from *Arabidopsis* causes specific changes in root growth and xylem differentiation. Plant J..

[77] Kudo T., Makita N., Kojima M., Tokunaga H., Sakakibara H. (2012). Cytokinin activity of cis-zeatin and phenotypic alterations induced by overexpression of putative cis-Zeatin-O-glucosyltransferase in rice. Plant Physiol..

[78] Vyroubalová S., Václavíková K., Turecková V., Novák O., Smehilová M., Hluska T., Ohnoutková L., Frébort I., Galuszka P. (2009). Characterization of new maize genes putatively involved in cytokinin metabolism and their expression during osmotic stress in relation to cytokinin levels. Plant Physiol..

[79] Havlová M., Dobrev P.I., Motyka V., Storchová H., Libus J., Dobrá J., Malbeck J., Gaudinová A., Vanková R. (2008). The role of cytokinins in responses to water deficit in tobacco plants over-expressing trans-zeatin O-glucosyltransferase gene under *35S* or *SAG12* promoters. Plant Cell Environ..

[80] Dobra J., Motyka V., Dobrev P., Malbeck J., Prasil I.T., Haisel D., Gaudinova A., Havlova M., Gubis J., Vankova R. (2010). Comparison of hormonal responses to heat, drought and combined stress in tobacco plants with elevated proline content. J. Plant Physiol..

[81] Schäfer M., Brütting C., Meza-Canales I.D., Grosskinsky D.K., Vankova R., Baldwin I.T., Meldau S. (2015). The role of cis-zeatin-type cytokinins in plant growth regulation and mediating responses to environmental interactions. J. Exp. Bot..

[82] Silva-Navas A., Conesa C.M., Saez A., Navarro-Neila S., Garcia-Mina J.M., Zamarreño A.M., Baigorri R., Swarup R., del Pozo J.C. (2019). Role of cis-zeatin in root responses to phosphate starvation. New Phytol..

[83] Miyawaki K., Tarkowski P., Matsumoto-Kitano M., Kato T., Sato S., Tarkowska D., Tabata S., Sandberg G., Kakimoto T. (2006). Roles of *Arabidopsis* ATP/ADP isopentenyltransferases and tRNA isopentenyltransferases in cytokinin biosynthesis. Proc. Natl. Acad. Sci. USA.

[84] Burbulis I.E., Iacobucci M., Shirley B.W. (1996). A null mutation in the first enzyme of flavonoid biosynthesis does not affect male fertility in *Arabidopsis*. Plant Cell.

[85] Liu Y.J., Jiang H., Zhao Y., Li X., Dai X.L., Zhuang J.H., Zhu M.Q., Jiang X.L., Wang P.P., Gao L.P. (2019). Three *Camellia sinensis* glutathione S-transferases are involved in the storage of anthocyanins, flavonols, and proanthocyanidins. Planta.

[86] Chen Y.X., Chen Y.S., Shi C.M., Huang Z.B., Zhang Y., Li S.K., Li Y., Ye J., Yu C., Li Z. (2018). SOAPnuke: A MapReduce acceleration-supported software for integrated quality control and preprocessing of high-throughput sequencing data. Gigascience.

[87] Kim D., Landmead B., Salzberg S.L. (2015). HISAT: A fast spliced aligner with low memory requirements. Nat. Methods.

[88] Wang J.B., Zhang Q.L., Tung J.F., Zhang X., Liu D., Deng Y.T., Tian Z.D., Chen H.L., Wang T.T., Yin W.X. (2024). High-quality assembled and annotated genomes of *Nicotiana tabacum* and *Nicotiana benthamiana* reveal chromosome evolution and changes in defense arsenals. Mol. Plant.

[89] Li B., Dewey C.N. (2011). RSEM: Accurate transcript quantification from RNA-Seq data with or without a reference genome. BMC Bioinform..

[90] Love M.I., Huber W., Anders S. (2014). Moderated estimation of fold change and dispersion for RNA-seq data with DESeq2. Genome Biol..

[91] Kanehisa M., Goto S., Kawashima S., Okuno Y., Hattori M. (2004). The KEGG resource for deciphering the genome. Nucleic Acids Res..

[92] Kanehisa M. (2002). The KEGG database. Novartis Found. Symp..

[93] Xie C., Mao X.Z., Huang J.J., Ding Y., Wu J.M., Dong S., Kong L., Gao G., Li C.Y., Wei L.P. (2011). KOBAS 2.0: A web server for annotation and identification of enriched pathways and diseases. Nucleic Acids Res..

[94] Zhao P., Zhou X.M., Zhao L.L., Cheung A.Y., Sun M.X. (2020). Autophagy-mediated compartmental cytoplasmic deletion is essential for tobacco pollen germination and male fertility. Autophagy.

[95] Zhang S.S., Ying H., Pingcuo G.S., Wang S., Zhao F., Cui Y.N., Shi J., Zeng H., Zeng X.L. (2019). Identification of potential metabolites mediating bird’s selective feeding on *Prunus mira* flowers. Biomed. Res. Int..

[96] Chen W., Gong L., Guo Z.L., Wang W.S., Zhang H.Y., Liu X.Q., Yu S.B., Xiong L.Z., Luo J. (2013). A novel integrated method for large-scale detection, identification, and quantification of widely targeted metabolites: Application in the study of rice metabolomics. Mol. Plant.

[97] Thévenot E.A., Roux A., Xu Y., Ezan E., Junot C. (2015). Analysis of the human adult urinary metabolome variations with age, body mass index, and gender by implementing a comprehensive workflow for univariate and OPLS statistical analyses. J. Proteome Res..

[98] Chong J., Xia J.G. (2018). MetaboAnalystR: An R package for flexible and reproducible analysis of metabolomics data. Bioinformatics.

[99] Cai W.J., Ye T.T., Wang Q., Cai B.D., Feng Y.Q. (2016). A rapid approach to investigate spatiotemporal distribution of phytohormones in rice. Plant Methods.

