# Supplementary material for: Transcriptome and Metabolome Analyses Reveal a Complex Stigma Microenvironment for Pollen Tube Growth in Tobacco
Source: Int J Mol Sci. 2024 Nov 14;25(22):12255. doi: 10.3390/ijms252212255 (PMC11594504; doi:10.3390/ijms252212255)
Supplement: Supplementary file 1 [file ijms-25-12255-s001.zip › Supplementary Materials.pdf]

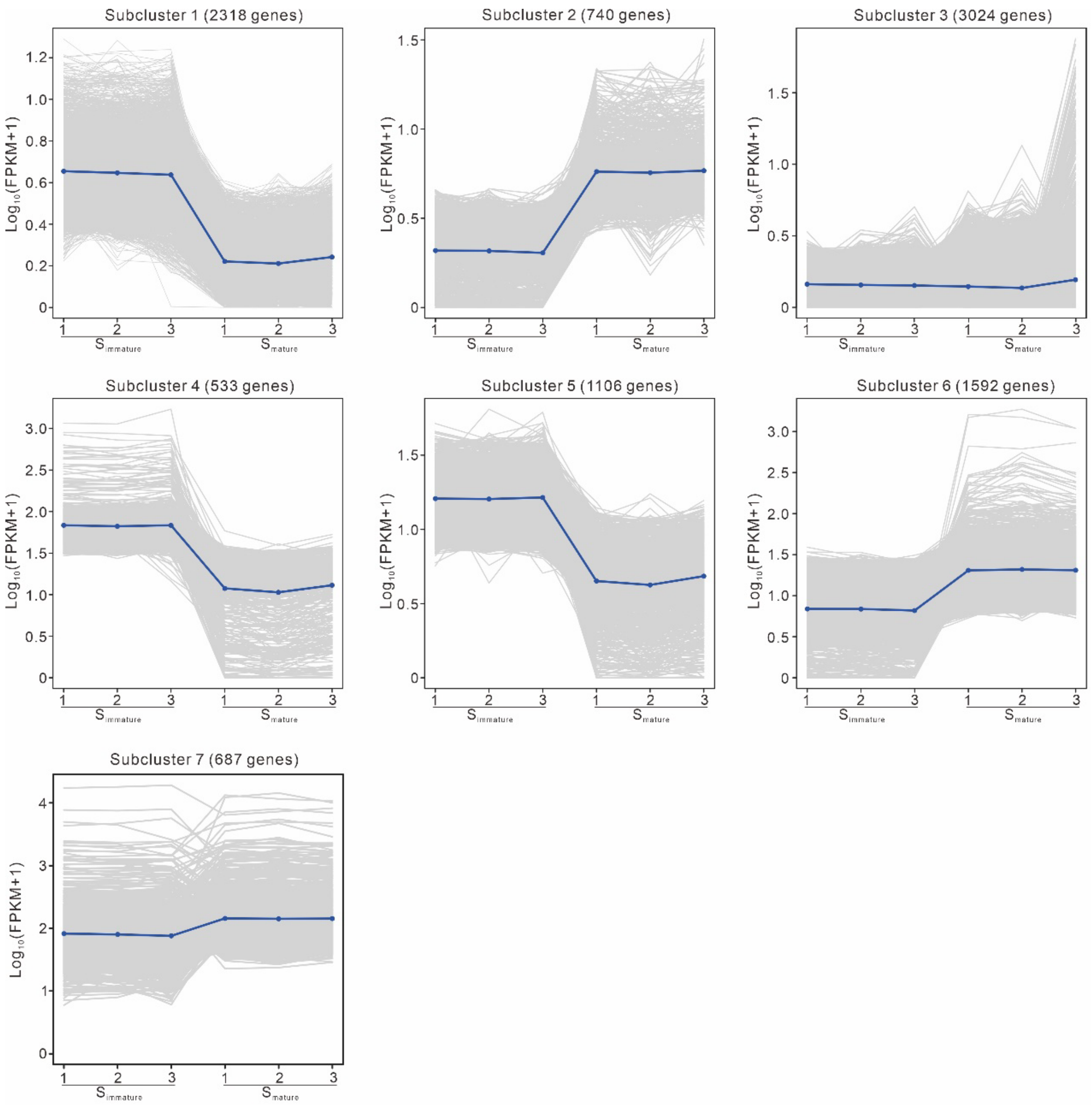

Figure S1. Expression profiles of differential expressed genes between $S_{immature}$ and S_{mature}. The mean expression level of all the genes in each cluster is plotted in blue line.

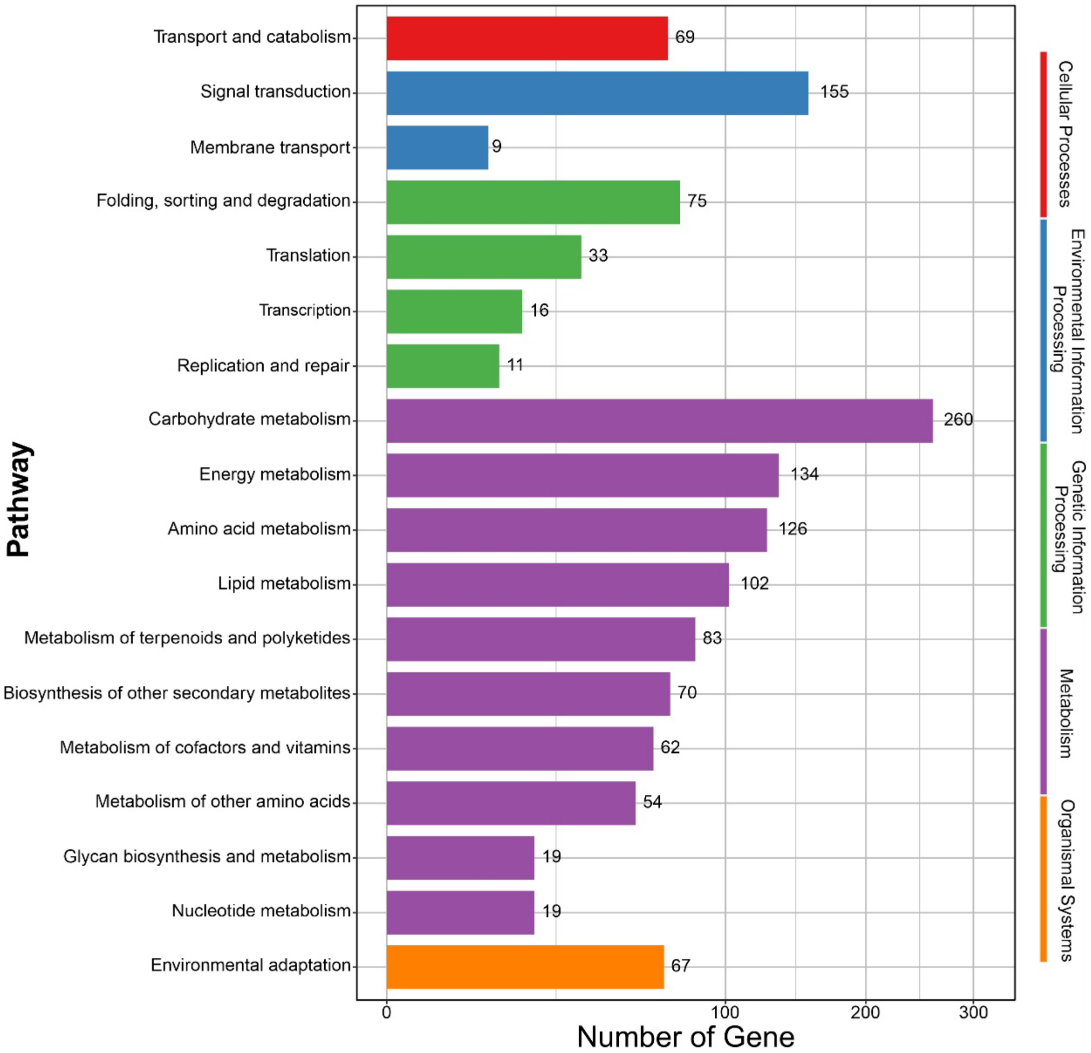

Figure S2. KEGG pathway analysis of upregulated genes in S_{mature}. The number of enriched genes is shown above the column.

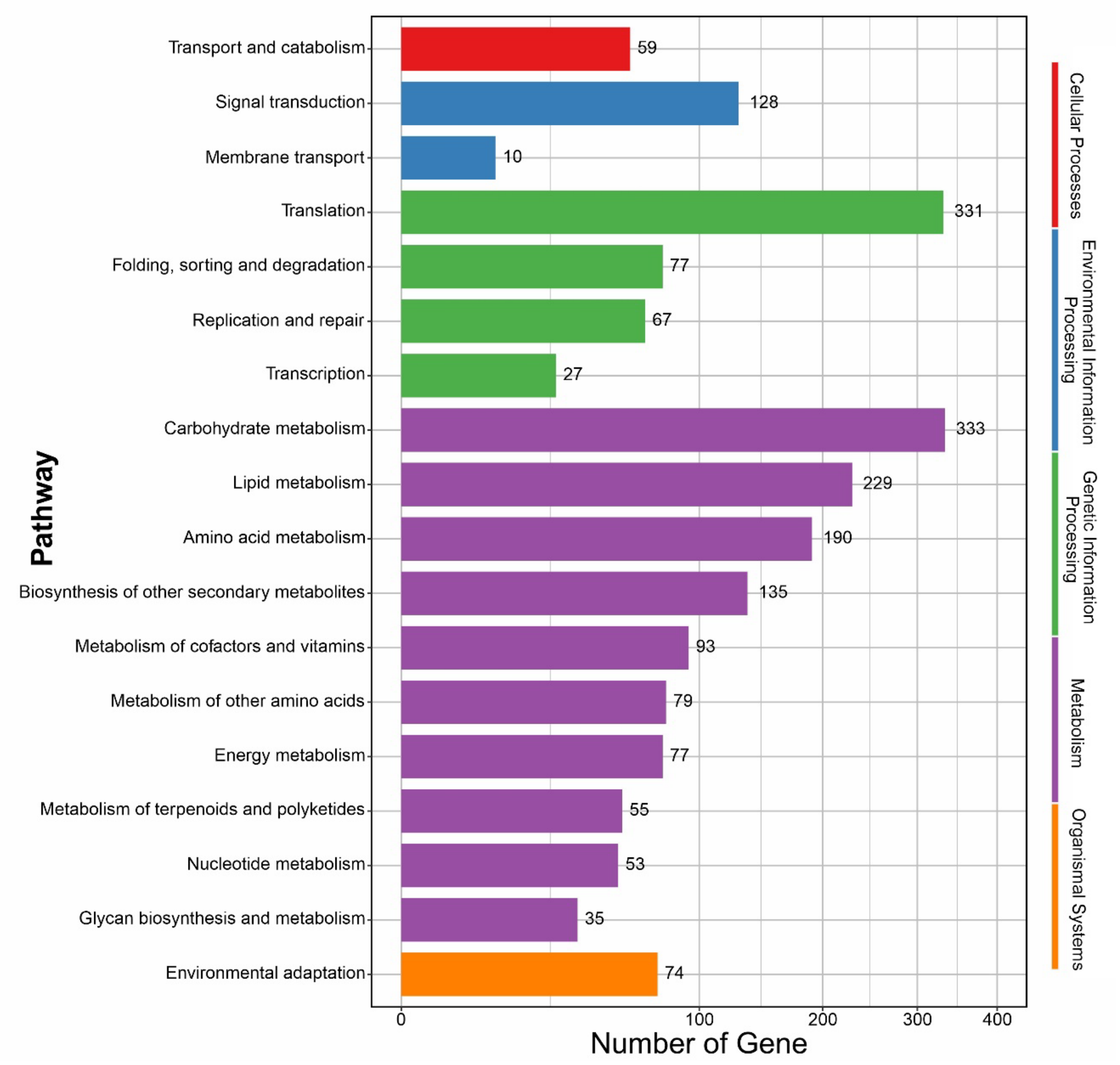

Figure S3. KEGG pathway analysis of downregulated genes in S_{mature}. The number of enriched genes is shown above the column.

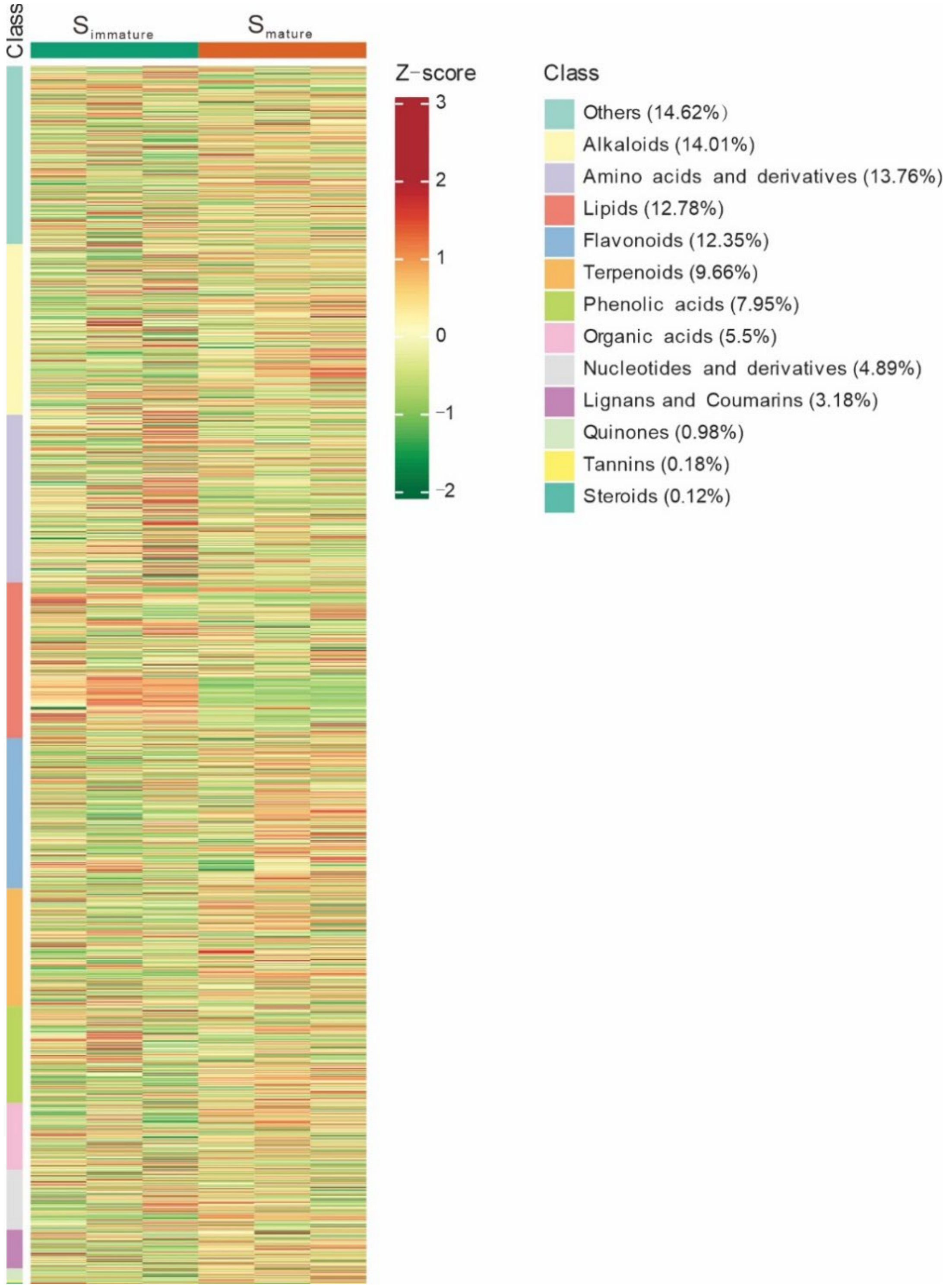

Figure S4. Heat maps displaying the relative metabolite contents from immature and mature stigmas.

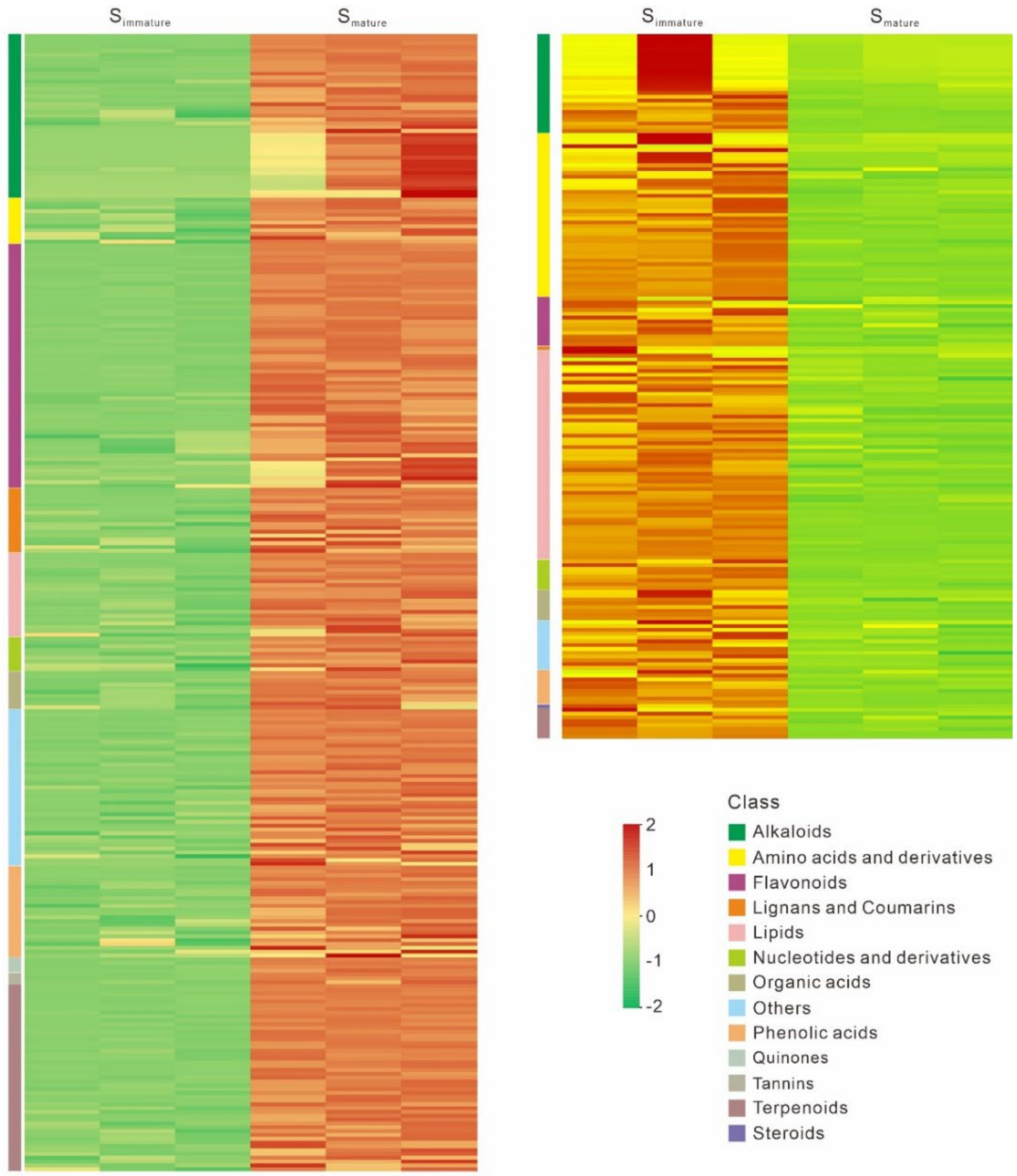

Figure S5. Heat maps displaying the up- and down-regulated metabolites between $S_{immature}$ and S_{mature}. See details in Supplementary Data S5.

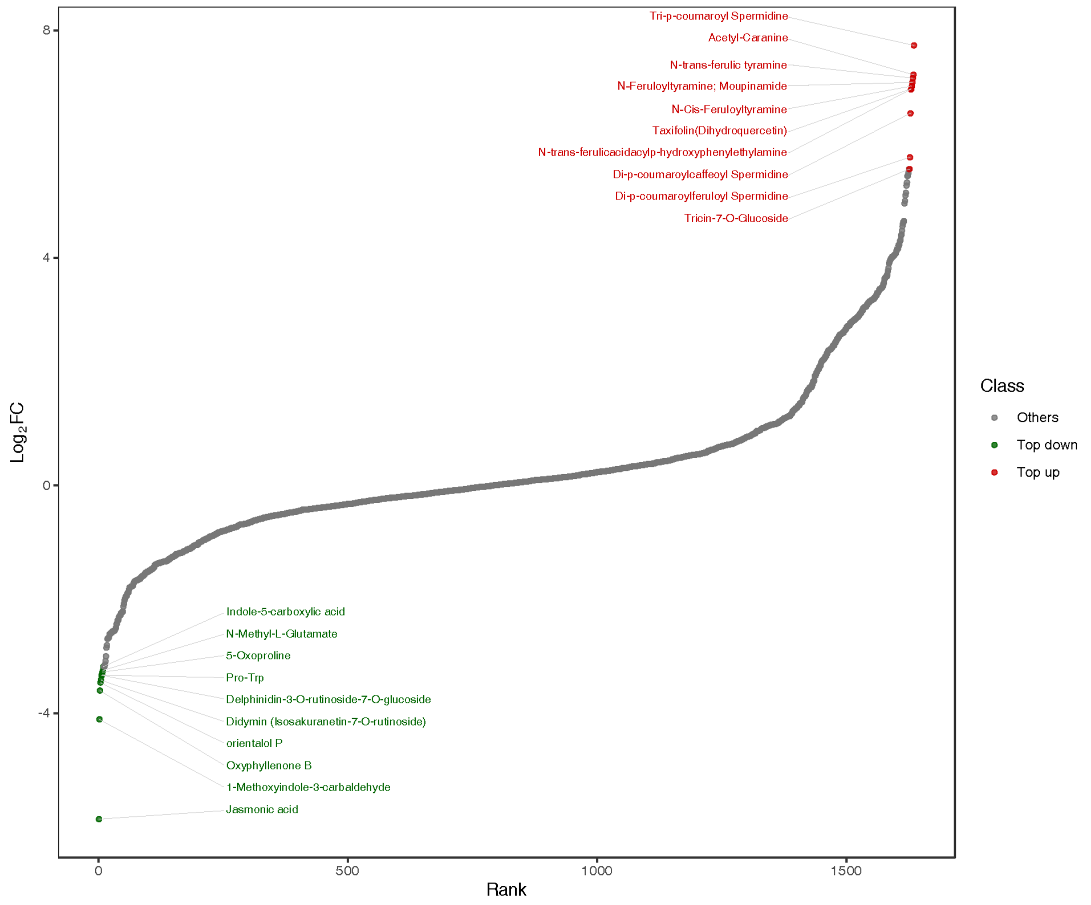

Figure S6. The top 20 differentially accumulated metabolites between immature and mature stigmas, with top 10 up-regulated metabolites shown as red dots and top 10 down-regulated metabolites shown as green dots.

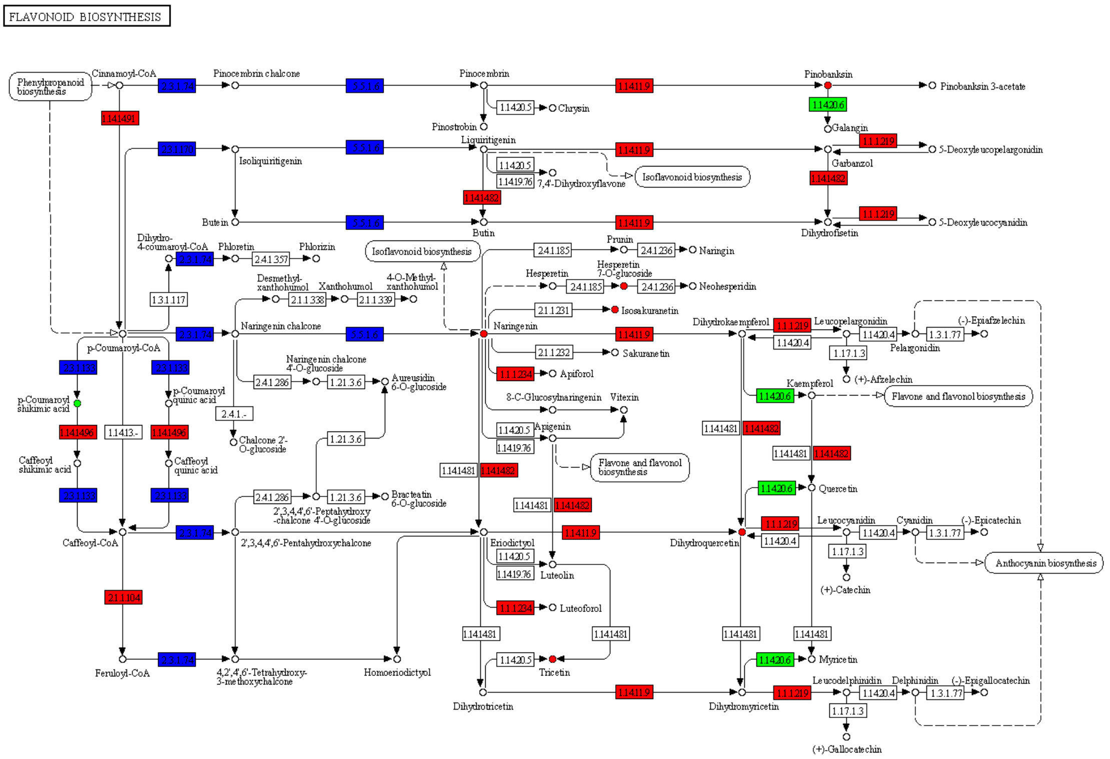

Figure S7. Differentially expressed genes and accumulated metabolites enriched in the flavonoid biosynthesis pathway (Ko00941, cited from Kanehisa laboratories). Small circles and boxes represent metabolites and genes respectively. Red indicates up-regulated, green indicates down-regulated and blue has both up- and down-regulated in Smature.

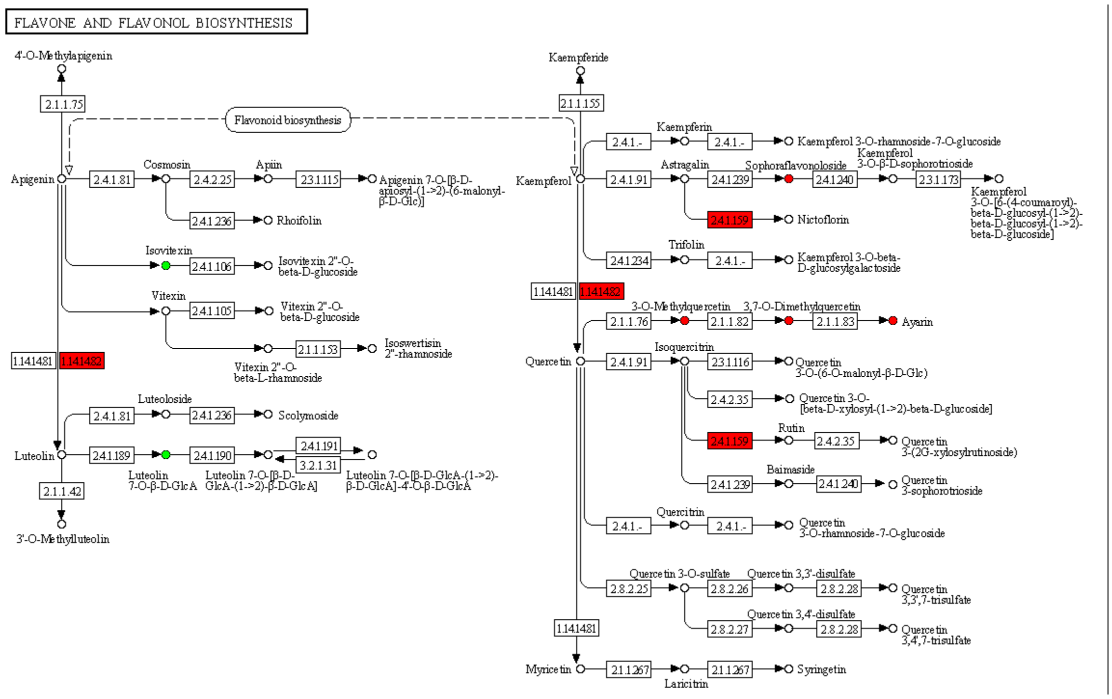

Figure S8. Differentially expressed genes and accumulated metabolites enriched in the flavone and flavonol biosynthesis pathway (Ko00944, cited from Kanehisa laboratories). Small circles and boxes represent metabolites and genes respectively. Red indicates up-regulated, green indicates down-regulated and blue has both up- and down-regulated in S_{mature}.

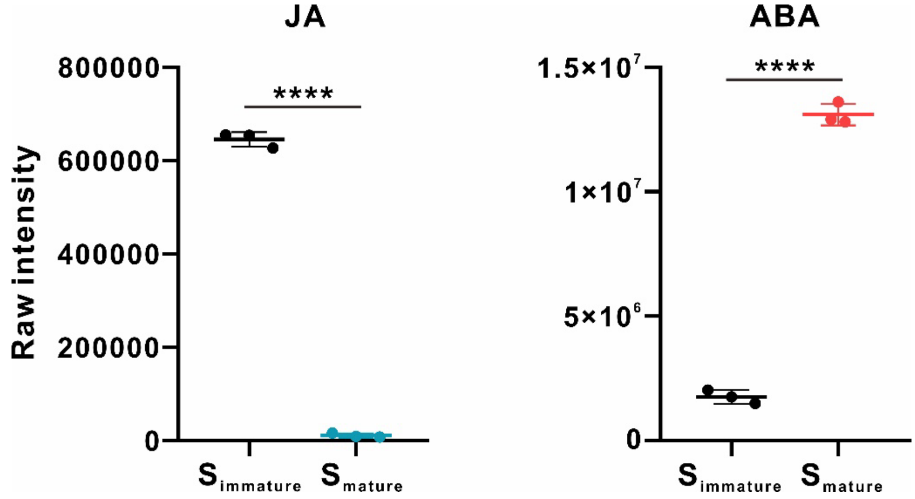

Figure S9. Differentially accumulated JA and ABA between immature and mature stigmas through metabolite profiling. Data are the mean ± SD from three independent replicates. Two-tailed Student's t test was used for statistical analysis (****$P < 0.0001$). ABA, Abscisic acid; JA, Jasmonic acid.
